# Multi-environment ecogenomics analysis of the cosmopolitan phylum Gemmatimonadota

**DOI:** 10.1128/spectrum.01112-23

**Published:** 2023-09-21

**Authors:** Izabela Mujakić, Pedro J. Cabello-Yeves, Cristian Villena-Alemany, Kasia Piwosz, Francisco Rodriguez-Valera, Antonio Picazo, Antonio Camacho, Michal Koblížek

**Affiliations:** 1 Laboratory of Anoxygenic Phototrophs, Institute of Microbiology of the Czech Academy of Sciences, Třeboň, Czechia; 2 Department of Ecosystem Biology, Faculty of Science, University of South Bohemia, České Budějovice, Czechia; 3 Cavanilles Institute of Biodiversity and Evolutionary Biology, University of Valencia, Paterna, Valencia, Spain; 4 Evolutionary Genomics Group, Departamento de Producción Vegetal y Microbiología, Universidad Miguel Hernández, San Juan de Alicante, Alicante, Spain; 5 School of Life Sciences, University of Warwick, Coventry, United Kingdom; 6 Department of Fisheries Oceanography and Marine Ecology, National Marine Fisheries Research Institute, Gdynia, Poland; Institut Ruder Boskovic, Zagreb, Croatia

**Keywords:** Gemmatimonadota, gemmatimonadetes, anoxygenic phototrophs, MAGs, metagenome, RuBisCO

## Abstract

Gemmatimonadota is a diverse bacterial phylum commonly found in environments such as soils, rhizospheres, fresh waters, and sediments. So far, the phylum contains just six cultured species (five of them sequenced), which limits our understanding of their diversity and metabolism. Therefore, we analyzed over 400 metagenome-assembled genomes (MAGs) and 5 culture-derived genomes representing Gemmatimonadota from various aquatic environments, hydrothermal vents, sediments, soils, and host-associated (with marine sponges and coral) species. The principal coordinate analysis based on the presence/absence of genes in Gemmatimonadota genomes and phylogenomic analysis documented that marine and host-associated Gemmatimonadota were the most distant from freshwater and wastewater species. A smaller genome size and coding sequences (CDS) number reduction were observed in marine MAGs, pointing to an oligotrophic environmental adaptation. Several metabolic pathways are restricted to specific environments. For example, genes for anoxygenic phototrophy were found only in freshwater, wastewater, and soda lake sediment genomes. There were several genomes from soda lake sediments and wastewater containing type IC/ID ribulose-1,5-bisphosphate carboxylase/oxygenase (RuBisCO). Various genomes from wastewater harbored bacterial type II RuBisCO, whereas RuBisCO-like protein was found in genomes from fresh waters, soil, host-associated, and marine sediments. Gemmatimonadota does not contain nitrogen fixation genes; however, the *nosZ* gene, involved in the reduction of N_2_O, was present in genomes from most environments, missing only in marine water and host-associated Gemmatimonadota. The presented data suggest that Gemmatimonadota evolved as an organotrophic species relying on aerobic respiration and then remodeled its genome inventory when adapting to particular environments.

**IMPORTANCE:**

Gemmatimonadota is a rarely studied bacterial phylum consisting of a handful of cultured species. Recent culture-independent studies documented that these organisms are distributed in many environments, including soil, marine, fresh, and waste waters. However, due to the lack of cultured species, information about their metabolic potential and environmental role is scarce. Therefore, we collected Gemmatimonadota metagenome-assembled genomes (MAGs) from different habitats and performed a systematic analysis of their genomic characteristics and metabolic potential. Our results show how Gemmatimonadota have adapted their genomes to different environments.

## INTRODUCTION

The bacterial phylum Gemmatimonadota was established in 2003 when the type species, *Gemmatimonas aurantiaca*, was isolated from a wastewater treatment plant (1). Since then, only five more species have been described. *“Gemmatirosa kalamazoonesis*,” *Roseisolibacter agri*, and *Longimicrobium terrae* were isolated from various soils (2 – 4), while *Gemmatimonas phototrophica* and *Gemmatimonas groenlandica* originated from fresh waters (5, 6). Due to the low number of cultured species, our understanding of the metabolic properties of Gemmatimonadota is very limited. All isolates grow on liquid organic carbon media under aerobic or semi-aerobic conditions (7, 8). In addition, two cultured freshwater species are facultative photoheterotrophs. They perform anoxygenic phototrophy and can supplement their metabolism with light energy harvested using bacteriochlorophyll (BChl)-*a*-containing photosystems; however, they require a supply of organic substrate for growth (5, 9, 10). Photoheterotrophic Gemmatimonadota, similar to Proteobacteria, have their photosynthesis genes organized in the photosynthesis gene cluster, containing *bch* and *crt* genes encoding enzymes of bacteriochlorophyll and carotenoid synthesis, *puf* and *puh* operons encoding the subunits of reaction centers and light-harvesting complexes, and various regulatory genes (6, 9, 11, 12).

Metagenomic analyses have documented that Gemmatimonadota is present in a wide range of environments (13, 14). They are one of the most abundant phyla in soils, representing on average 2% of 16S rRNA gene sequences (13, 15, 16), and are relatively common in fresh waters, where they typically constitute 1% of bacteria but may contribute even up to 9% of the bacterial community (7, 12, 17, 18). Gemmatimonadota were found in soda lake sediments, where they represented ≥1% of 16S rRNA gene sequences (19). Only minimum numbers have been registered in the marine water column (20), and typically in marine environments, they are found associated with sponges (21, 22), deep-sea hydrothermal vents (23, 24), or sediments (25, 26), where they represent up to 2.4% of the total bacterial 16S rRNA reads (27).

Previously, we documented a high diversity of photoheterotrophic Gemmatimonadota in freshwater lakes (12). Interestingly, metagenome-assembled genomes (MAGs) containing genes for both anoxygenic photosynthesis and carbon fixation were identified in soda lake sediments (28, 29). However, there is only limited information about Gemmatimonadota inhabiting other environments, such as soils or marine waters. Therefore, we analyzed all publicly available MAGs (up until 3 May 2021) affiliated with Gemmatimonadota to get a global picture of their metabolic functions, patterns, and genomic differences across multiple environments. In addition, we assembled 16 MAGs from four Spanish freshwater reservoirs (Tables S1 and S2). We focused on key metabolic pathways, such as carbon assimilation, nitrogen and sulfur cycles, and photoheterotrophic capability, to define the potential roles of Gemmatimonadota in nutrient cycling and to decipher the specific differences in their physiology based on the environment from which they originate.

## RESULTS AND DISCUSSION

### Basic characteristics of the Gemmatimonadota genomes

**FIG 1:**
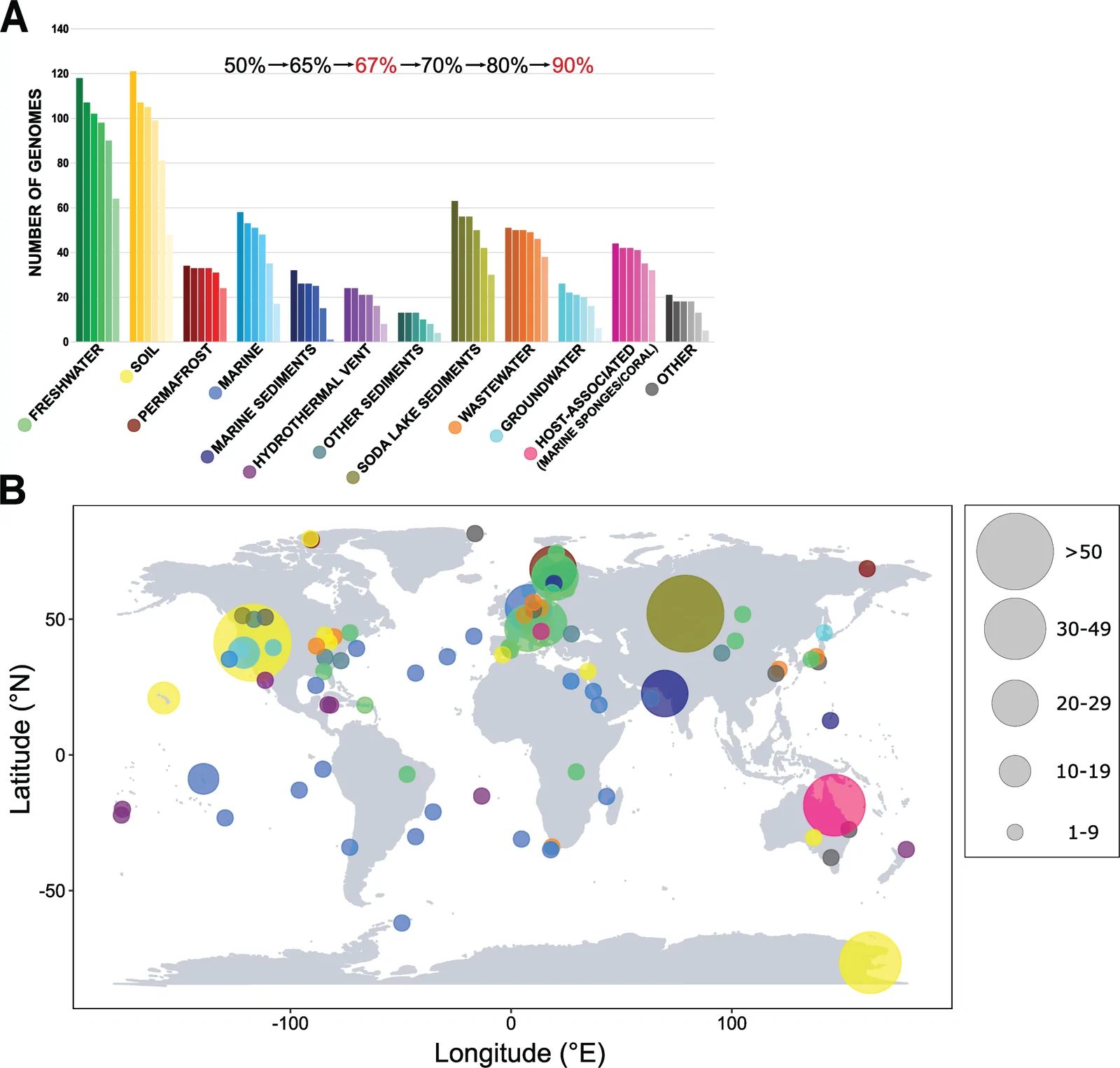
Distribution of Gemmatimonadota in different environments. (**A**) Bar plot showing the number of Gemmatimonadota genomes present in the NCBI database, including newly assembled freshwater MAGs, divided based on the environment of origin and completeness. Each bar represents the number of genomes with different completeness levels, from MAGs with more than >50% up to >90% completeness. The completeness levels used in subsequent analyses are marked in red. (**B**) Map showing where the MAGs used in our analyses originated from. Environments are color coded, and the size of the circle represents the number of MAGs obtained from the specific location.

Gemmatimonadota MAGs were classified based on their environmental origin in 12 different categories (Fig. 1A and B). The numbers of dereplicated genomes within each category were as follows: fresh waters 91, soils 90, wastewaters 49, soda lake sediments 46, marine waters 42, host-associated (i.e., associated with marine sponges and coral) 25, permafrost 22, marine sediments 12, hydrothermal vents 18, groundwater 21, and other sediments 13. The final category “Other” consisted of 13 genomes from varying environments and was not included in most analyses (unless stated otherwise). Genomes from all environments varied largely in size (1.68–7.77 Mbp), with an average of 3.59 Mbp and 2,744 coding sequences (CDS) (Fig. S1A and B). The smallest genomes (1.68–3.01 Mbp) with the lowest number of genes and higher homogeneity were those from marine waters. Genomes from potentially more nutrient-rich environments, like soils, soda lake sediments, marine sediments, and wastewaters, had larger sizes as well as a higher number of CDS (Fig. S2A). This is consistent with previous studies documenting that nutrient limitation affects genome size, GC content, or coding density (30 – 32). The average coding density was 92.9% (84%–97%), and despite the high variability, it was on average higher in MAGs from fresh waters and soils than in those from marine waters, marine sediments, or wastewaters (Fig. S1C). The average median intergenic distance was 35.45 bp. Even though marine genomes are in general smaller, they have on average longer intergenic spacers than freshwater, soil, or wastewater genomes (Fig. S1D and S2B). Genomes from soda lake sediments and marine sediments have both larger sizes and longer median intergenic spacers. Lengths of intergenic spacers vary substantially among bacteria (32) and often contain regulatory elements with key functions (33).

**FIG 2:**
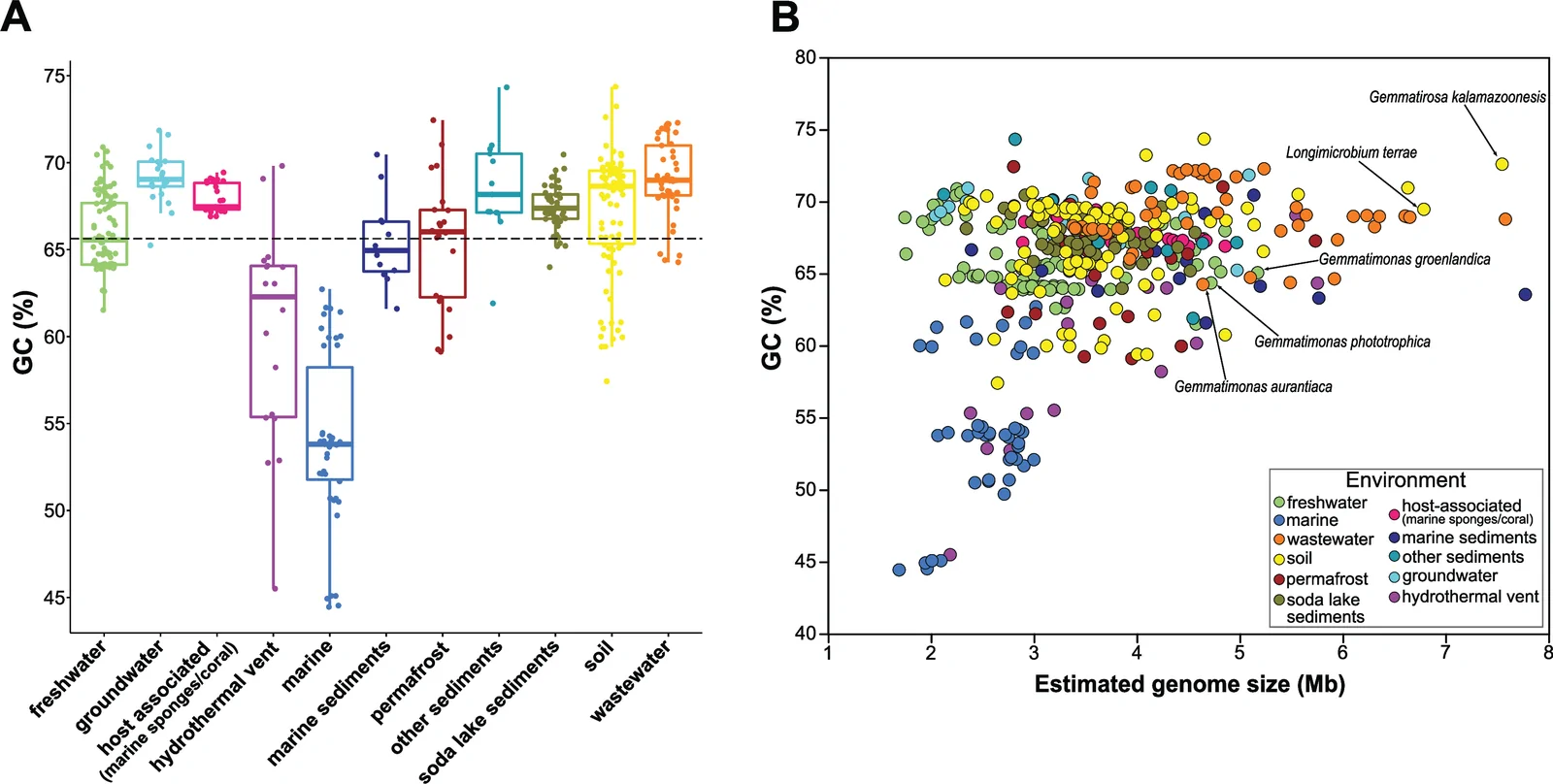
(A) Distribution of GC content (%) of Gemmatimonadota genomes based on their environmental origin. (**B**) A comparison of estimated genome size and GC content (%) of Gemmatimonadota genomes from different environments. Genomes are color-coded based on their environment. Labels depict the cultured Gemmatimonadota species.

The GC content in the studied genomes ranged from 44.4% to 74.4%, with an average of 65.6% (Fig. 2A and B). Marine genomes and several others from hydrothermal vents had the lowest GC content (range 44.4%–62.7% and 45.5%–69.8%, respectively). The GC content of bacterial communities is known to be influenced by the environment (34), and low GC content among marine bacteria is a common phenomenon (35) interpreted as an adaptation to low nitrogen (36) or a result of evolutionary history (37). It must be noted that the distribution of the GC content in marine genomes was trimodal (44%–45%, 49%–54%, and 59%–62.7%), indicating an additional sub-environmental division of genomes from the same origin, possibly depending on parameters such as the water depth or water nutrient concentration. However, the associated metadata in the NCBI did not contain enough details to fully explain this pattern. The influence of environment on the GC content of bacterial communities can be observed even in closely related species, which in different environments show significant differences in GC content (34). Freshwater genomes were also smaller but had a higher GC content than marine genomes (Fig. 2A). Gemmatimonadota genomes from other environments like soil, permafrost, or wastewater varied in genome sizes and had on average a higher GC content than marine MAGs, a trait common for bacteria living in more nutrient-rich environments (38). This, combined with their larger genomes and higher number of CDS, indicates their higher metabolic potential and advantages under different environmental conditions.

### Gemmatimonadota habitat-related core and accessory gene analysis

We explored the main shared (core) and flexible (accessory) genomes among Gemmatimonadota MAGs with >90% completeness and <10% contamination across multiple origins. Generally, the size of the habitat-dependent core and flexible genome of Gemmatimonadota differed between environments and ranged from a lower average of 2,677 genes in the marine environment (10 genomes), 2,868 in the freshwater environment (29 genomes), to the highest average of 4,659 genes in the wastewater environment (13 genomes) (Table S3), indicating how contrasting environments differentially shape their gene inventories. Larger genomes found in wastewater may encode a wider variety of enzymes for utilization in an environment often enriched with nutrients (38, 39). The size of the shared genes (strict core and soft core) also varied (Fig. S3), while accessory genes formed by the shell (40) and cloud (41) categories represented more than 50% of the flexible/accessory genome in all environments except marine waters, showing high variability in the gene inventories among the members of the phylum.

### Multi-environment principal coordinate and phylogenomic analyses

**FIG 3:**
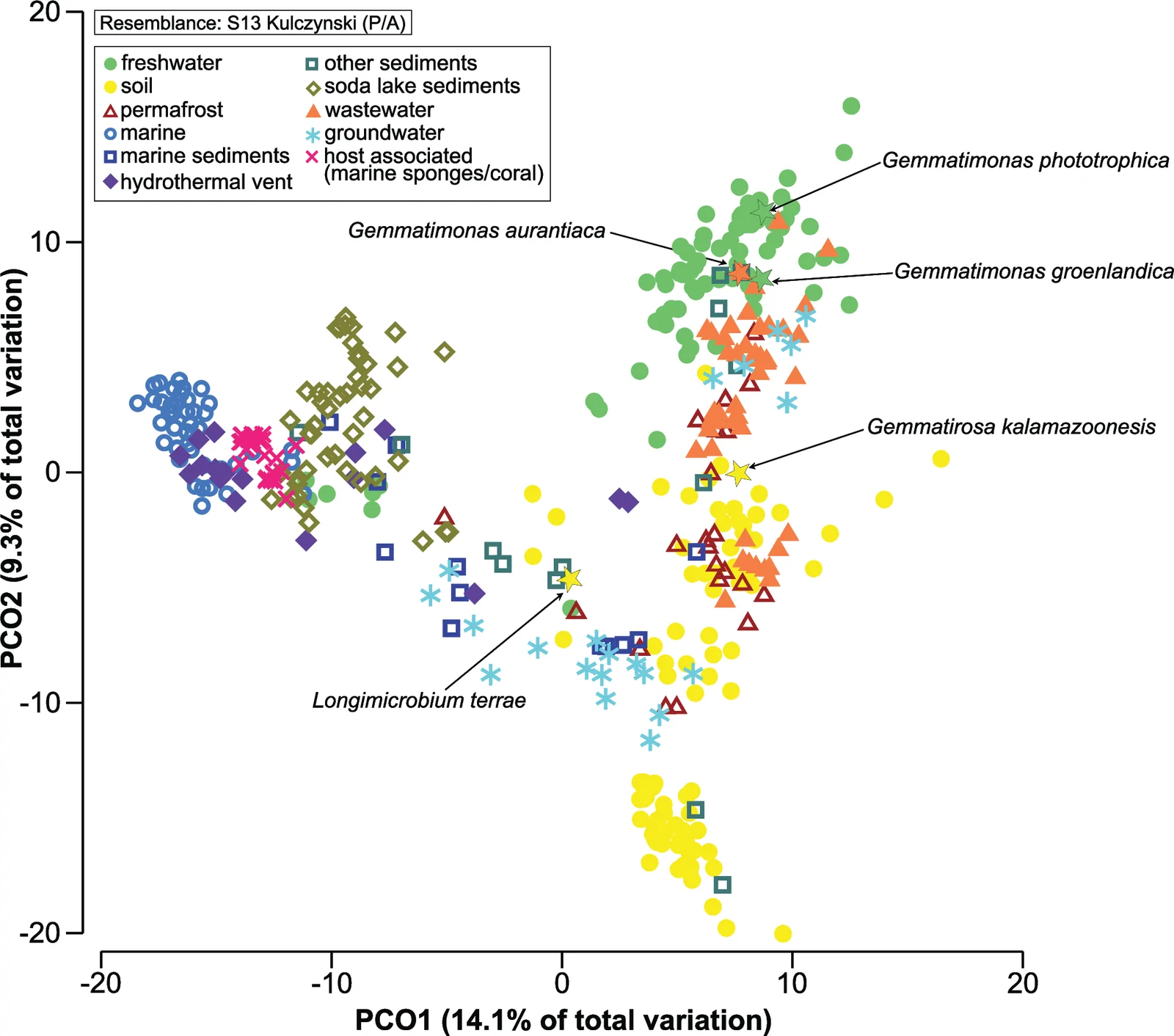
PCoA using Kulczynski resemblance matrix based on SEED presence/absence of the genes in Gemmatimonadota genomes, showing grouping of genomes based on their environment. The legend in the upper left corner shows that environments are color coded and a different symbol is assigned to each environment. Cultured Gemmatimonadota species are labeled and shown with a star symbol.

The similarity among genomes was studied using a principal coordinate analysis (PCoA) based on the presence or absence of genes (Table S4). The genomes clustered based on their environmental origin (Fig. 3), indicating their differential adaptation to specific environments. Permutational multivariate dispersion (PERMDISP) analysis documented a significant difference in heterogeneity levels (PERMDISP, *P* < 0.05) between various environments (Table S5), and significant differences in gene presence/absence were detected for Gemmatimonadota from all environments (PERMANOVA, *P* < 0.001) except marine sediments and other sediments (PERMANOVA, *P* = 0.019). Similarity percentage (SIMPER) analysis based on Bray-Curtis similarity showed host-associated (70.6%) and marine waters (69.9%) MAGs to be the most similar among them, while those from other sediments (56.2%), marine sediments (59.2%), and groundwater (59.7%) were the least similar (Table S5). In the comparison between different environments, marine MAGs were more like other marine-related environments such as hydrothermal vents or host-associated (with marine sponges and coral) (average dissimilarity of 37.79% and 40.21%, respectively), while freshwater MAGs were more similar to wastewater, groundwater, and permafrost MAGs (<41% of average dissimilarity). The highest dissimilarities (>47%) were seen between soil vs marine and host-associated (with marine sponges and coral) MAGs and between marine vs wastewater MAGs.

**FIG 4:**
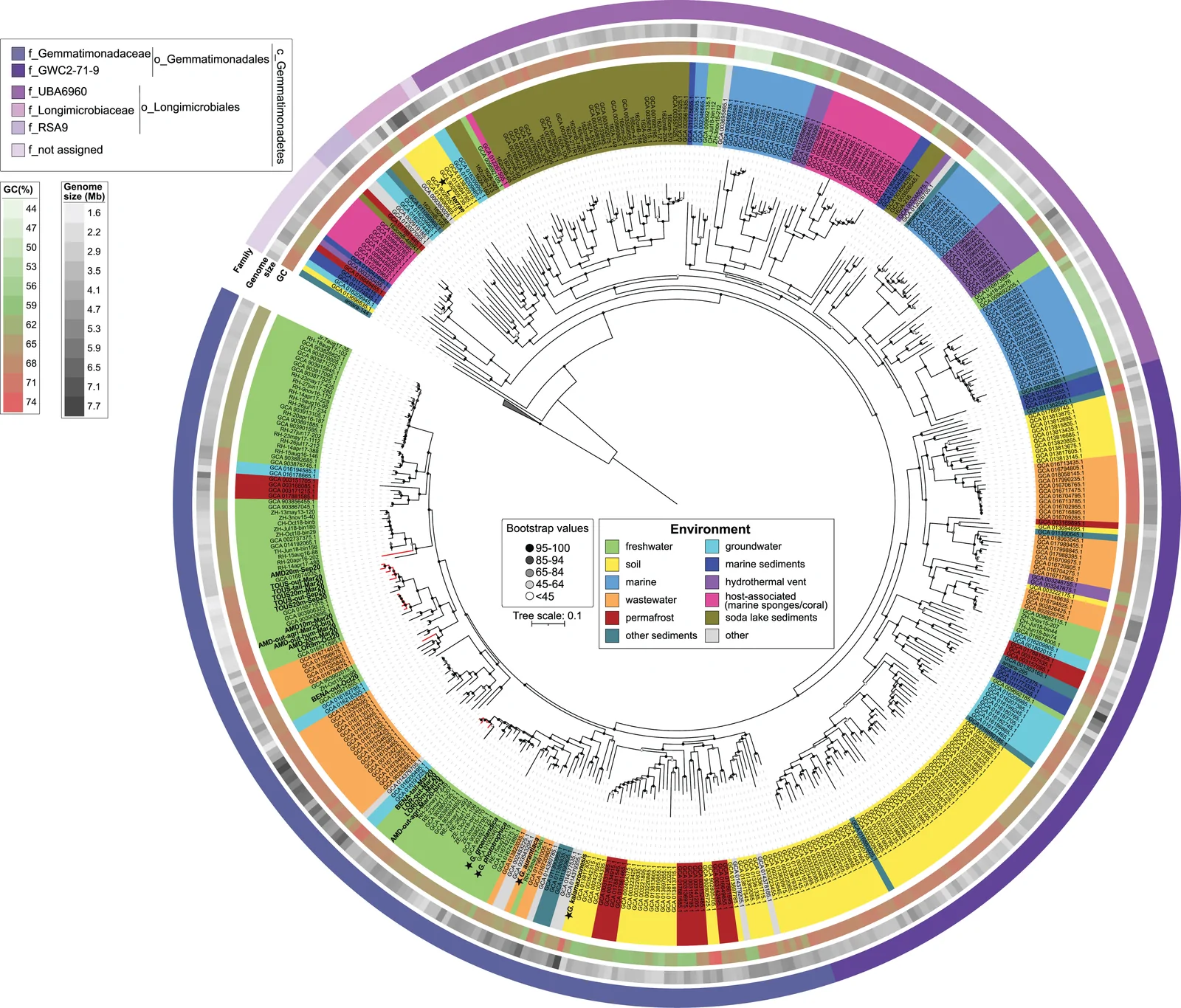
Phylogenomic tree of Gemmatimonadota genomes based on 400 universally conserved and most ubiquitous proteins present in the PhyloPhlAn database (42, 43). The collapsed branch represents an outgroup consisting of three genomes from the bacterial phylum Fibrobacterota (GCA_900142455.1 *Hallerella intestinalis*, GCA_900217845.1 *Fibrobacter elongatus*, GCA_000146505.1 *Fibrobacter succinogenes*). The strength of support for internal nodes is shown through gray-scale-colored circles (center legend). All genomes are color-coded based on their environmental origin (center legend). The following annotations, starting from innermost to outermost indicate GC content (%), estimated genome size (Mb), and family level classification. The legend for each outer circle is represented in the upper left corner. Details on all genomes can be found in Tables S1 and S2.

Similar patterns were found in the phylogenomic analysis (Fig. 4), albeit these genomes did not cluster exclusively according to the environment of origin. Most of the MAGs obtained belonged to two families inside the order Gemmatimonadales. The family Gemmatimonadaceae encompassed most of the MAGs from fresh, waste, and groundwater, along with genomes from permafrost and soil (Fig. S4), and cultured species *G. phototrophica*, *G. groenlandica*, *G. aurantiaca*, and *G. kalamazoonesis*. This family also contained all the MAGs from Spanish reservoirs reconstructed in this study. Eleven of them formed a clade related to MAGs from the hypolimnion of several Swiss lakes and Římov Reservoir (Czech Republic). The remaining four clustered together with a previously assigned group, Pg2 (12), which consists of freshwater phototrophic Gemmatimonadota from the epilimnion of Lake Zurich (Switzerland) and Římov Reservoir. The second family GWC2-71-9 mostly contained genomes from the soil, wastewater, permafrost, groundwater, and other sediments, with only a small number of genomes from freshwater lakes and marine sediments (Fig. S4).

The second largest group was formed by MAGs belonging to the order Longimicrobiales, which was established based on the soil bacterium *Longimicrobium terrae* (4). This order mostly contains marine water, marine sediments, hydrothermal vents, soda lake sediments, and host-associated (with marine sponges and coral) genomes, together with several genomes from the hypolimnion of deep freshwater lakes. This is in line with our previous observations that Gemmatimonadota from deep freshwater lakes are related to those from marine environments or environments like soil and sediments (12). Host-associated MAGs (marine sponges and corals) were part of two different families (Longimicrobiales and a not assigned family), and while they are closely related to marine water genomes, the differences in the gene repertoire between these two environments were significant (PERMANOVA, *P* < 0.0001), and they represent real symbionts of marine sponges and corals (44, 45).

### Main metabolic pathways across the Gemmatimonadota phylum

**FIG 5:**
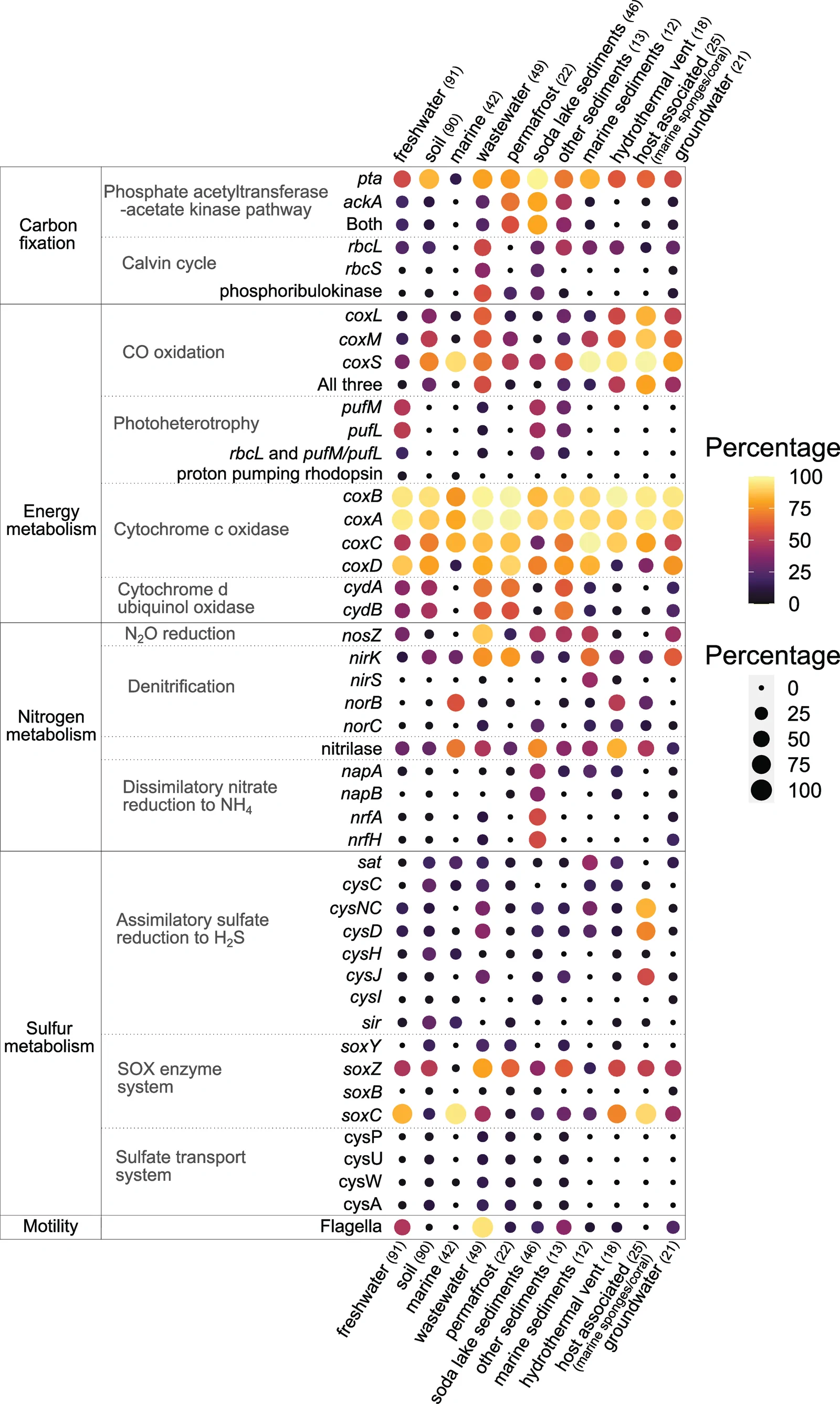
Bubble plot showing the percentages of key genes involved in specific pathways present in Gemmatimonadota genomes from different environments. Dot color and size indicate the percentage of each gene in any given environment, with the darkest color and smallest size of the dot marking the absence of said gene in that environment. The number of MAGs in each environment is labeled in parenthesis. Details about genes presence/absence can be found in Table S6.

**FIG 6:**
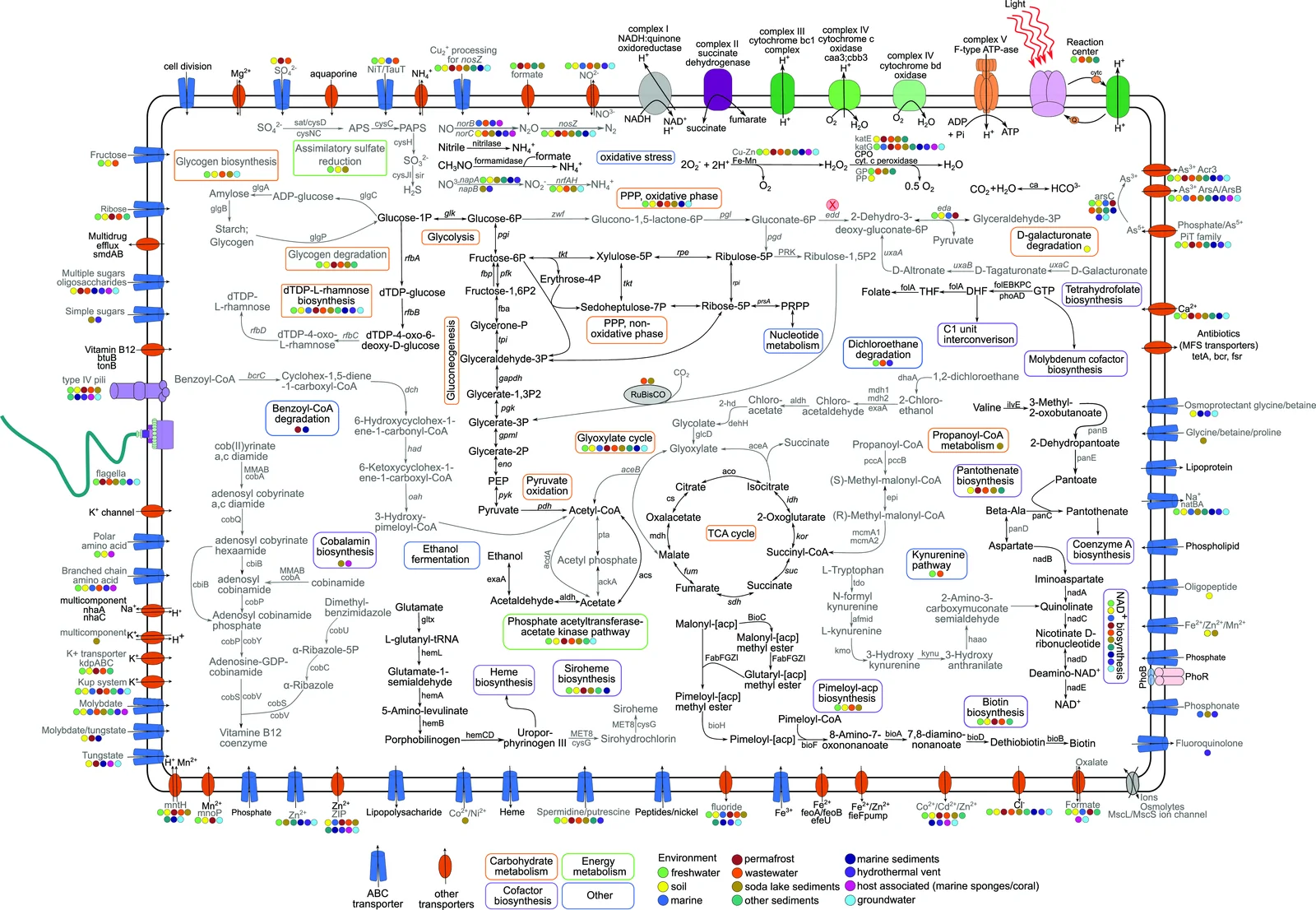
A metabolic reconstruction of Gemmatimonadota showing some of the key pathways. Four different colored rectangles depict the names of pathways and metabolic processes. Pathways labeled with black are present in all Gemmatimonadota genomes, while those labeled with gray are only found in Gemmatimonadota genomes from certain environments. Color-coded circles representing different environments of origin indicate that the said gene/pathway/transporter was present in that environment (shown if at least two genomes showed presence). Details of genomes can be found in Table S6. Abbreviations for compounds: PEP, phosphoenolpyruvate; PPP, pentose phosphate pathway; PRPP, 5-phosphoribosyl 1-pyrophosphate; CPO, chloroperoxidase; GP, glutathione peroxidase; PP, porphyrinogen peroxidase; THF, tetrahydrofolate; DHF, dihydrofolate; GTP, guanosine 5′-triphosphate; APS, adenylyl sulfate; PAPS, 3′-phosphoadenylyl sulfate; NAD^+^, nicotinamide adenine dinucleotide.

The core metabolism of the Gemmatimonadota phylum was reported recently (13). Still, both PCoA and phylogenomic analysis showed that their gene inventories vary depending on their origin, presumably due to adaptation to the specific conditions and selection pressure in any particular habitat. To study this further, we looked for the main metabolic commonalities and uniqueness associated with each environment. To do so, we individually inspected genomes from all environments to reconstruct a metabolic model of the Gemmatimonadota phylum with pathways present or absent for each environmental specialist (Fig. 5; Fig. S5; Fig. 6; Table S6).

#### Basic energy metabolism

Gemmatimonadota from all environments contained basic genes for respiratory metabolism such as NADH:quinone oxidoreductase, cytochrome *c* oxidase, F-type ATPase, subunits of succinate dehydrogenase involved in oxidative electron transfer chains, or enzymes of heme biosynthesis (Fig. 6). Cytochrome *bd* ubiquinol oxidase (encoded by *cydAB* genes) with high affinity for oxygen (46) was present in MAGs from most environments, except for marine water and host-associated ones. The host-associated genomes also lacked succinate dehydrogenase cytochrome *b* subunit (*sdhC*), while in marine water MAGs, it was present only in one genome. Genes encoding fumarate reductase, a key enzyme in anaerobic respiration that catalyzes the reduction of fumarate to succinate, were found in host-associated (60%), soda lake sediment (36.9%), hydrothermal vent (33.3%), marine sediment (8.3%), groundwater (14.3%), and marine water (7.14%) MAGs.

Gemmatimonadota also contained genes necessary for central carbohydrate metabolism, including glycolysis (Embden-Meyerhof pathway), gluconeogenesis, tricarboxylic acid cycle, coenzyme A biosynthesis, the aerobic route of oxidation of pyruvate to acetyl-CoA *via* pyruvate dehydrogenase, and the biosynthesis of phosphoribosyl diphosphate, which is needed to produce purines and pyrimidines (Fig. S5). The ED (Entner-Doudoroff) pathway to obtain pyruvate without glycolysis was reported as less common in Gemmatimonadota (13), and similarly, we saw that the key enzyme for this pathway, 2-dehydro-3-deoxyphosphogluconate aldolase (*eda*), was present in MAGs from soil and permafrost (52.2% [47 genomes] and 27.3% [6 genomes], respectively) and almost absent in MAGs from marine, fresh, and wastewaters; other sediments; and marine sediments, where it was found only in three genomes at most. Moreover, phosphogluconate dehydratase (*edd*), which catalyzes another key step in this pathway, was absent in MAGs from all environments. Alternatively, the ED pathway could be supplied through the degradation of D-galacturonate (13), which can be an important carbon source for microorganisms. The pathway for degradation of D-galacturonate was present in soil Gemmatimonadota (key enzymes were present from 18.8% to 54.4% of MAGs, with a complete pathway in 16.6% of MAGs) (Fig. S5).

Gemmatimonadota from all environments encoded representative genes for the non-oxidative phase of the pentose phosphate pathway, while both key enzymes for the oxidative phase (*zwf*, PGD) were predominately found in permafrost (72.7%), soil (47.7%), and marine sediment genomes (41.6%). A common feature was also the presence of genes encoding the biosynthesis of dTDP-L-rhamnose, an important cell wall component, except for the host-associated MAGs, which lacked two key enzymes (*rfbC* and *rfbD*). As these MAGs live in a symbiotic association, it is likely that they do not require these enzymes, as the same pathway seems to be also missing in Alphaproteobacteria associated with marine sponges (47).

Several metabolic pathways were only common in Gemmatimonadota from specific environments. For example, key enzymes of the glyoxylate cycle (isocitrate lyase [*aceA*] and malate synthase [*aceB*]) were found in soil genomes (52.2%), other sediments (30.7%), groundwater (28.5%), permafrost (22.7%), wastewater (18.4%), marine sediments (16.6%), marine waters (7.1%), soda lake sediments (4.3%), and fresh waters (3.3%). Bacteria harboring this pathway can assimilate acetate in the absence of complex substrates (48, 49). Moreover, while some of the genes encoding the pathway for conversion of propionyl-CoA to succinyl-CoA occurred in all environments, all key genes (Fig. S5) were present only in MAGs from soda lake sediments (21.7%).

Furthermore, MAGs from all environments showed the potential to degrade polysaccharides. The gene encoding endoglucanase (cellulase) was common among MAGs from all environments, while xylanase (endo-1,4-beta-xylanase, *xynA*) was present in soil (22.2%), permafrost (9.1%), and sporadically in freshwater (7.7%) and wastewater MAGs (2.04%). Chitinase (c*hiC*) was present in MAGs from wastewater (28.6%), permafrost (27.3%), marine sediments (25%), marine water (23.8%), soil (16.6%), as well as in several genomes of other sediments (7.7%) and fresh waters (8.8%). Additionally, MAGs from permafrost (59.1%), soil (58.8%), groundwater (47.6%), fresh waters (43.9%), hydrothermal vent (16.6%), and wastewater (6.1%) had chitin disaccharide deacetylase (*chbG*), which is suggested to catalyze the deacetylation of chitin, making it an easily degradable substrate (50).

One of the main storage molecules that helps bacteria survive periods when nutrients or energy sources are scarce is glycogen (51 – 54). The enzymes for its biosynthesis (1,4-alpha-glucan branching enzyme, glucose-1-phosphate adenylyltransferase, and glycogen synthase) were present in freshwater (21.9%), wastewater (18.3%), soil (11.1%), three groundwater, and soda lake sediment genomes. Similarly, the complete pathway for glycogen degradation was mostly found in wastewater (46.9%), other sediment (30.7%), permafrost (18.2%), freshwater (16.5%), and soil (8.8%) genomes. The ability of some bacteria to accumulate glycogen as an energy reserve (51) allows them to quickly activate their metabolism when nutrient availability increases, providing a competitive advantage in nutrient-fluctuating environments.

#### Carbon fixation strategies

Gemmatimonadota from several different environments contained genes encoding the large subunit of ribulose-1,5-bisphosphate carboxylase/oxygenase (RubisCO, *rbcL*), sometimes in two or three copies. There are three forms of RuBisCO (type I, II, and III) that catalyze the carboxylation and oxygenation of ribulose 1,5-bisphosphate (55). The most widespread type I was reported earlier in six Gemmatimonadota MAGs reconstructed from soda lake sediments (28, 29), which were included in this analysis. Genes encoding type I RuBisCO, phylogenetically identified as type IC/ID (Fig. S6), were present in soda lake sediments (28.3%), wastewater (36.7%), groundwater (4.7%), soil (3.3%), freshwater (1.1%), and one MAG from a glacier (Other). Most of these MAGs also contained genes encoding the small subunit of RuBisCO (*rbcS*) and phosphoribulokinase (Fig. 5). None of the MAGs contained the proteobacterial, α-cyanobacterial (IA), or β-cyanobacterial form (IB) of RuBisCO (56).

Bacterial type II RuBisCO, which is less efficient in discriminating between CO_2_ and O_2_ and adapted to environments with low oxygen concentrations (55, 57), was present in wastewater MAGs (36.7%). Type II is commonly found in Proteobacteria (58) and organisms that also have type I (55), as is the case for some of the wastewater MAGs (12.2%), which had both types. In addition, 72 MAGs contained the so-called type IV *rbcL* gene, which is probably not involved in carbon fixation (55, 59, 60).

Key genes encoding the phosphate acetyltransferase-acetate kinase pathway for carbon fixation, in which acetate produced from acetyl-CoA can be used as a carbon source or electron donor, were found in soda lake sediment (80.4%), permafrost (59.1%), other sediment (38.4%), wastewater (24.5%), freshwater (19.8%), and soil MAGs (11.1%). In the freshwater environment, the presence of the two key genes was observed in two photoheterotrophic species as well as in four MAGs from Spanish reservoirs.

Carbon monoxide (CO), an atmospheric trace gas, can be an alternative energy source for some organoheterotrophic bacteria during organic carbon starvation, enhancing their survival (61 – 63). The gene encoding the large subunit of carbon monoxide dehydrogenase (*coxL*) is highly abundant in soils where it can facilitate atmospheric CO removal and was reported to be present in soil Gemmatimonadota (61, 64). In this study, we found the c*oxL* gene as well as the genes for small (*coxS*) and medium (*coxM*) subunits of carbon monoxide dehydrogenase in host-associated (80%), wastewater (57.1%), hydrothermal vent (50%), groundwater (42.8%), soil (30%), other sediment (23.1%), marine sediments (16.6%), permafrost (9.1%), and freshwater (5.5%) MAGs.

#### Phototrophy

Many freshwater Gemmatimonadota are aerobic anoxygenic phototrophic (AAP) species (6, 11, 12). A common marker gene for AAP bacteria is the *pufM* gene, which encodes the M subunit of the bacterial photosynthetic reaction center (65). Here, we identified genes encoding type II photosynthetic reaction centers (*pufM* and/or *pufL*) in 51.6% of all freshwater MAGs (Fig. 5), as well as in MAGs from soda lakes (47.8%), other sediments (30.7%), wastewater (16.3%), and group Other (glacier [3 MAGs], biofilm [1 MAG]), while they were absent from all other environments. Interestingly, 28.3% of phototrophic MAGs from soda lake sediments and 8.2% from wastewater also contained type I *rbcL*, indicating that these species may have the potential for photoautotrophic growth (28, 29).

Many aquatic microorganisms harvest light energy using proton-pumping rhodopsins (66, 67). However, among Gemmatimonadota, this system is very rare. We found genes encoding green- or blue-light-absorbing proteorhodopsins only in five genomes, which originated from deeper layers of freshwater lakes Baikal, Constance, Zurich, and Biwa. A xanthorhodopsin gene was identified in one marine and two glacier MAGs (category Other). One of the MAGs from the glacier also contained genes for BChl-*a*-based photoheterotrophy, indicating the potential for dual phototrophy (68).

#### Nitrogen cycle

Nitrogen metabolism in Gemmatimonadota is relatively simple. No nitrogen fixation genes were found in any of the analyzed MAGs, which means that Gemmatimonadota must rely on combined nitrogen sources such as ammonium or amino acids. The gene encoding high-affinity ammonium transporter (Amt), a preferred nitrogen source for microbial growth, was present in MAGs from all environments, as well as the gene encoding nitrilase that hydrolyzes nitriles to ammonia. Branched-chain amino acid transporters were a common feature for host-associated, marine, hydrothermal vent, and wastewater genomes, while spermidine/putrescine or nitrate-nitrite/taurine transporters were more common in wastewater, freshwater, and soil genomes (Fig. 6).

The complete denitrification pathway was not identified in any of the analyzed MAGs. However, the nitrous oxide reductase (*nosZ*) gene was found in genomes from all environments except marine water and host-associated (Fig. 5). This enzyme catalyzes the final step of denitrification (69 – 72) but is also considered an independent respiratory reaction since it is often found in organisms lacking other genes for denitrification, such as *nirK*, *nirS*, and *nor* (73). In Gemmatimonadota MAGs, the *nirK* gene (NO^−^-forming nitrite reductase) was found in all environments; however, the *nirS* gene was only present in marine sediments and single genomes from hydrothermal vents and wastewater. Gemmatimonadota *nosZ* genes seem to be one of the most abundant in soil environments (74 – 76), and their high presence in other environments points to their potentially important role in reducing the N_2_O. Both *G. aurantiaca* and *G. kalamazoonesis* have been suggested to use N_2_O as a substitute for O_2_ to survive temporary anoxia during transitions between oxic and anoxic states, which can be common in soil or wastewater environments (71, 77). Furthermore, 59.5% of marine, 44.4% of hydrothermal vent, and 28% of host-related MAGs did not have *nosZ* but contained *norB* (nitric oxide reductase subunit B), which converts nitric oxide to N_2_O and could point to their genetic potential to produce N_2_O. The presence of this gene in host-associated Gemmatimonadota suggests their potential role in nitrogen cycling as part of the marine sponge microbiome (44). Finally, genes for dissimilatory nitrate reduction to ammonia (*napAB* and *nrfAH*) were common in soda lake sediments (28), probably due to the anaerobic conditions that can occur in these habitats.

#### Sulfur cycle

Regarding the sulfur cycle, the distribution of genes encoding enzymes involved in assimilatory sulfate reduction to H_2_S (*sat*, *cysC*, *cysNC*, *cysD*, *cysH*, *cysJ*, *cysI,* and sulfite reductase) was patchy (Fig. 5). In this pathway, sulfate is reduced to H_2_S, which is then incorporated into cysteine, which can be subsequently used for the synthesis of other sulfur-containing molecules (78, 79). The complete pathway was found in the highest numbers in soil (18.9%) and soda lake sediments (10.8%). In fresh water, hydrothermal vents, permafrost, and host-associated environments, it was only present in up to three genomes. Genes encoding for the sulfate transport system, which enables sulfate-sulfur assimilation, were mostly found in wastewater MAGs, with a lower occurrence in soil and permafrost MAGs. Furthermore, the complete sox enzyme system, involved in thiosulfate oxidation to SO_4_^2−^, was not found in any Gemmatimonadota genomes, although they contained some genes (*soxZ*, *soxY*, *soxC*, or *soxB*), depending on the environment.

#### Phosphate

Phosphate is one of the main biogenic elements required for the biosynthesis of nucleic acids and lipids. Due to its low natural availability, it is the limiting nutrient in many natural environments. The main route for its uptake in Gemmatimonadota from all environments was the high-affinity phosphate transport system (*pstSCAB*), and they could regulate its acquisition through the PhoR-PhoB two-component system. Additionally, marine (19%), two MAGs from soda lake sediments, and hydrothermal vents had an uptake system for phosphonate (*phnCDE*), a good source of phosphorus under phosphate starvation (80). During phosphorus starvation, many bacteria can produce alkaline phosphatases (*phoA*, *phoX*, *phoD*), which catalyze hydrolysis of phosphoesters (81, 82). *PhoA* was found in Gemmatimonadota from all environments, with the lowest numbers in host-associated MAGs (8%) and the highest in permafrost MAGs (68.2%). In contrast, *phoX* was present in up to two MAGs in marine sediment and fresh waters and was generally found in lower numbers in all environments except for host-associated (92%), marine water (57.1%), and wastewater (55.1%). Finally, *phoD* was highly present in freshwater (92.3%), wastewater (69.4%), and hydrothermal vent (66.6%) MAGs. The polyphosphate kinase gene used for the accumulation of polyphosphate was present in all Gemmatimonadota MAGs except marine. The presence of all these genes, which are crucial during phosphorus limitation, as well as the high-affinity phosphate transport system, indicates that Gemmatimonadota has different strategies to cope with phosphorus limitations.

#### Protection against oxidative stress

Gemmatimonadota is composed of mostly aerobic organisms that depend on aerobic respiration. Therefore, their genomes encode many proteins involved in the protection from oxidative damage and stress that is associated with an aerobic lifestyle (Fig. 6). [Fe-Mn] and [Cu-Zn] families of superoxide dismutases were present in MAGs from all environments, except for marine and hydrothermal vents, where the [Cu-Zn] family was not found. Cytochrome *c* peroxidase also occurred in all environments, while glutathione peroxidase was found in high numbers in wastewater (53.1%) and fresh water (38.4%), and in other environments was present only a in a few representatives or not at all. Catalase peroxidase *katG* was present in all MAGs except soil, while catalase *katE* was found in soda lake sediments (39.1%), other sediments (23%), wastewater (22.4%), permafrost (18.2%), soils (12.2%), and only one freshwater bacterium, *G. groenlandica* (6). From other types of peroxidases, chloroperoxidase occurred in all environments, while porphyrinogen peroxidase was only present in two MAGs from soil and one from soda lakes and marine sediments.

So far, all cultured Gemmatimonadota contain large amounts of carotenoids. These pigments protect cells from excess light as well as against reactive oxygen species, and in AAP species, they can act as additional light-harvesting pigments (11, 83). Gemmatimonadota MAGs from wastewaters (85.7%, 67.3%), fresh waters (58.2%, 63.7%), other sediments (69.2%, 76.9%), soda lake sediments (39.13%, 91.3%), and soils (24.4%, 21.11%) contained genes encoding the initial parts for carotenoid biosynthesis, phytoene synthase (*crtB*) and phytoene dehydrogenase (*crtI*), respectively. They were almost absent in marine, permafrost, marine sediment, hydrothermal vent, and groundwater genomes, where they were found in up to two genomes. Host-associated genomes contained the *crtI* gene (24%), but *crtB* was present only in one genome. Other carotenoid biosynthesis genes found were β-carotene ketolase (*crtO*), present in all environments; lycopene beta-cyclase (*crtY*), found in a small number of soil and freshwater MAGs; up to three genomes of wastewaters, permafrost, marine water, marine sediments, and soda lake sediments; and β-carotene 3-hydroxylase (*crtZ*), found only in several freshwater and wastewater genomes.

#### Cofactors and vitamins

Gemmatimonadota cultures require a mixture of vitamins like biotin (vitamin B_7_), folic acid (vitamin B_9_), nicotinic acid (vitamin B_3_), pantothenic acid (vitamin B_5_), and cobalamin (vitamin B_12_) for growth (1 – 5). All analyzed MAGs contained the complete pathway for molybdenum cofactor synthesis. Molybdenum is a cofactor in numerous enzymes in prokaryotic and eukaryotic organisms (84). Folate biosynthesis could be inferred as complete in all environments, given that marine, host-associated, wastewater, and other sediment MAGs that lack one of the key enzymes (*folA*) have the gene encoding *thyX*, suggested to function as *folA* (85). Furthermore, MAGs from most environments encode genes involved in pantothenate biosynthesis, a precursor of coenzyme A, an essential molecule in metabolism. The exceptions were marine, freshwater, and host-associated MAGs, which lacked one of the key enzymes (*panD*). Genes encoding the biosynthesis of biotin, an essential cofactor of enzymes involved in fatty acid synthesis or amino acid metabolism (86), were present mostly in freshwater, wastewater, permafrost, other sediment, and several soil MAGs. In other environments, several genes for biotin biosynthesis were missing. Genes for NAD^+^ biosynthesis, an important metabolite and cofactor involved in nucleotide synthesis, were only sporadically present in host-associated MAGs, probably due to their incompleteness. Another biosynthetic pathway, the kynurenine pathway that leads to quinolinate, a precursor of NAD (87), was present in freshwater and wastewater MAGs. Soda lake sediment and host-associated MAGs had several genes involved in the late steps of cobalamin biosynthesis from cobyrinate *a*,*c*-diamide; however, genes involved in both aerobic and anaerobic cobalamin pathways were not found. This suggests that these genes may be used in the salvage pathway as a more effective way for obtaining cobalamin since the *btuB* transporter and *tonB* protein, which function together in cobalamin transport (88), were present in Gemmatimonadota.

#### Other genes

Genes for flagellar assembly were present in almost all wastewater (93.8%) and nearly half of freshwater (47.3%) MAGs (Fig. 5). This included five of the Gemmatimonadota MAGs from Spanish reservoirs and many limnic and planktonic MAGs from Římov Reservoir (12). Additionally, the presence of flagella was already shown for both freshwater cultures *G. phototrophica* and *G. groenlandica* (8). Smaller numbers were found in other sediments (38.4%), groundwater (23.8%), soda lake sediments (19.6%), and permafrost (13.6%). Host-associated and marine MAGs did not contain flagellar genes, while in soil, hydrothermal vents, and marine sediments, they only occurred in one or two genomes, respectively. Additionally, genomes from all environments but marine had genes encoding type IV pili (Fig. 6).

Some Gemmatimonadota also display different enzymes for degrading alkanes, which they may potentially use as a source of carbon and energy (89). Alkanes are naturally found in environments from sources like decaying microorganisms, algae, or plants but are also present in high content in crude oil, which can be a contaminant for the environment (89). The gene encoding alkanesulfonate monooxygenase (*alkB*), which degrades short alkanes (90), was found in the highest numbers in soil (30%), wastewater (18.4%), freshwater (14.3%), and marine MAGs (14.3%). Alkane-1-monooxygenase (*alkM*), used in the degradation of longer alkanes (90), was less common and was missing from most environments except marine (45.2%), host-associated (36%), and freshwater (8.8%) MAGs. The presence of these genes could suggest a potentially ecologically relevant role in the biodegradation of hydrocarbons in Gemmatimonadota. This potential for biodegradation is also evident in Gemmatimonadota MAGs from permafrost and marine sediments, which have genes involved in the degradation of benzoyl-CoA, a central intermediate of synthetic aromatic compounds (91). Moreover, Gemmatimonadota from wastewater, fresh water, hydrothermal vents, and one genome from marine sediments seems to be able to degrade 1,2- dichloroethane (13), an industrially produced pollutant in aquatic environments (92). Furthermore, Gemmatimonadota seems to utilize glycolate, converting it to glyoxylate, as they have genes encoding glycolate oxidase, a protein complex that consists of three subunits D, E, and F (*glcDEF*). This could explain the previous observation of a close association of limnic Gemmatimonadota with phytoplankton in freshwater environments (12), since glycolate is one of the most common cyanobacterial and algal exudates that can be utilized by bacteria (93 – 95). Additionally, the *glc* operon contains malate synthase G (*glcB*) that further converts glyoxylate to malate, which is then used for energy production in the TCA cycle (96).

Several different antimicrobial compounds and multidrug transport systems were present in all Gemmatimonadota, while importers and efflux systems of ions like Fe^2+^, Mg^2+^, Mn^2+^, Zn^2+^, and other heavy metals were present in different environments (Fig. 6). The Na^+^/H^+^ antiporter system to remove Na^+^ from cells, as well as Kch voltage-gated K^+^ channels, important in all prokaryotes for maintaining cellular homeostasis (31, 97), were present in all Gemmatimonadota. Trk-type, fast but low-affinity K^+^ transporter (97) predominated in marine water, marine sediment, and hydrothermal vent genomes, while K^+^ channels and the K^+^/H^+^ antiporter system predominated in MAGs from soda lake sediments, freshwater, soil, permafrost, and wastewater. To deal with hypo-osmotic stress, Gemmatimonadota from all environments had aquaporins, water channels that ease the water stress by enabling fast water efflux (98), and two types of mechanosensitive channels, MscL and/or MscS, which also helped cells return to normal, isotonic size (99).

### Conclusions

We have explored a large data set of Gemmatimonadota MAGs to characterize their metabolic potential in different environments. Phylogenomics and gene content analyses indicated that Gemmatimonadota have diverse and flexible metabolisms and the ability to adapt to different conditions. A common feature of all MAGs was aerobic organoheterotrophy, but many pathways were specific to some environments. For instance, photoheterotrophy and motility (flagella) were more prevalent in freshwaters, soda lakes, and wastewaters, whereas CO oxidation was more common in soils, marine sediments, hydrothermal vents, host-associated, and groundwater. Differences between environments could also be observed in their genomes’ sizes and GC content. The size and GC content of marine MAGs were the lowest, which is a common adaptation of marine bacteria to oligotrophic conditions. Moreover, Gemmatimonadota exhibit different strategies for survival under phosphorus limitation, some of which are present in all genomes, and some, like the uptake system for phosphonate, are more common in marine and hydrothermal vent genomes, or different alkaline phosphatases like *phoD* or *phoX* are more common in host-associated, freshwater, or wastewater genomes. Gemmatimonadota are unable to fix nitrogen; however, a potential environmental role in the reduction of N_2_O is highlighted by the presence of *nosZ* genes in all Gemmatimonadota except for marine, host-associated, and hydrothermal vents, in which the presence of *norB* could suggest that they may rather produce N_2_O. Finally, pathways for the degradation of synthetic solvents and aromatic compounds found in some Gemmatimonadota point to their potential role in biodegradation in the environment, and the ability to utilize glycolate indicates a potential symbiotic relationship with phytoplankton.

## MATERIALS AND METHODS

### Sampling, sequencing, and assembly

Samples collected from four Spanish freshwater reservoirs (Amadorio, Tous, Benageber, and Loriguilla) (56) were reused in this analysis. All four monomictic reservoirs are located in the semi-arid eastern region of Spain, close to the Mediterranean Sea. Briefly, for each reservoir, samples were taken in two campaigns (March—winter mixing period and September/October—summer stratification period in 2020) at three locations: the dam (epilimnion, deep chlorophyll maximum-DCM, and hypolimnion in summer; and epilimnion and hypolimnion in winter), the outlet of the reservoir, and tailwaters (0.5 m). In all cases, water samples were filtered through a series of 20, 5, and 0.22-µm filters, and DNA was extracted as described in reference (56). DNA extracted from 0.22-µm filters was sequenced with Illumina NovaSeq.

Metagenomes were assembled with IDBA-UD (100), which resulted in approximately 5,000 contigs >5 kb per metagenome, which were used for further binning. Binning was conducted using METABAT2 (101), and we obtained a total of 16 MAGs ascribed to the Gemmatimonadota phylum (Table S1). A quality check of 16 MAGs was made using the CheckM v1.1.3 package (102). All MAGs had <5% contamination, while completeness ranged from the lowest 67.79% to 100%.

### Analyzed data set

The obtained MAGs from the Spanish reservoirs were expanded with five cultured representatives and all publicly available MAGs of Gemmatimonadota from NCBI (downloaded on 3 May 2021) together with previously published freshwater Gemmatimonadota (12). All genomes (731) (Table S2) were checked for completeness and contamination using the CheckM package (102), and 68 MAGs with completeness below 50% and/or contamination above 10% were removed from further analysis. Moreover, basic metadata such as the environmental origin, assembly size, estimated genome size, GC content, median intergenic spacer, and coding density were obtained for each genome. Environmental origin and assembly size were collected from NCBI; GC content, median intergenic spacer, and coding density were calculated using the in-house pipeline; and estimated genome size was calculated based on the formula: (total sequence length/completeness) × (100 − contamination). All of these were taxonomically classified with the Genome Taxonomy Database (GTDB-Tk) with default settings (103). This identified 57 ambiguous genomes, which were re-classified as Latescibacterota and several other closely related bacterial phyla (e.g., Eisenbacteria, Krumholzibacteriota). These genomes were also removed from all subsequent analyses. Finally, in order to avoid bias and reduce redundancy, the remaining genomes were dereplicated using dRep v 2.3.3 (104), with parameters: -comp 50 -pa 0.99 -sa 0.995. The final data set consisted of 442 MAGs (completeness >67%, a value chosen because it represents 2/3 of the genome) that were divided based on their environmental origin into 12 different categories: freshwater, soil, marine water, marine sediment, hydrothermal vent, permafrost, soda lake sediments, other sediments, host-associated (with marine sponges and coral), wastewater, groundwater, and Other. The latter category (Other) included MAGs from glaciers (6), biofilm (1), bioreactors (2), fossil (1), compost (1), hot springs (2), and an unknown metagenome (1) (Table S2). Since the environments in this category were too diverse to be considered together, the MAGs were excluded from all of the analyses except for phylogenomics of Gemmatimonadota genomes, the RuBisCO tree, and phototrophy in metabolic analysis. Coding density plots showing comparison of estimated genome size with percent GC, number of CDS, and median intergenic spacers (bp) were performed for all genomes that had >67% completeness, excluding the category Other. The graphs were plotted using SigmaPlot v.14.0 and Rstudio v.3.6.1 (package ggplot2). The map in Fig. 1 was made in R Studio v.3.6.1 (package Maps), using data from Natural Earth, supported by NACIS (North American Cartographic Information Society) and free for use. All the graphs were edited in Inkscape v.1.0.

### Analysis of core and accessory genes of Gemmatimonadota from different habitats

The analysis of core and accessory genes from Gemmatimonadota genomes was done using the GET_HOMOLOGUES package based on diamond blastp and OMCL algorithms with default parameters (105). Only environments where MAGs/cultured genomes had completeness above 90% were analyzed. These included the following categories: soil (34), freshwater (29), soda lake sediments (20), host-associated (14), wastewater (13), marine (10), and permafrost (9). To avoid bias due to redundant genomes and variability in the completeness of similar MAGs, genomes with ≥98% average nucleotide identity in the same environment were excluded. This analysis must be treated with caution due to the varying level of completeness of the analyzed MAGs regardless of the environment where they originated and the differing numbers of MAGs used for each environment. The average number of core, soft core, shell, and cloud genes was calculated for each of these environmental groups (Table S3). Core genes were defined as being present in all considered genomes of the analyzed environment, and soft core genes were defined as being present in 95% of them. The shell category comprises moderately conserved genes present in <90% of compared genomes. Finally, cloud genes are rare genes present in only one or two genomes (105).

### PCoA/clustering plots and phylogenies

A principal coordinate ordination analysis with SEED (106) gene presence/absence (Table S4) was conducted for all genomes (excluding the category Other), with completeness higher than 67%. Briefly, a Kulczynski resemblance matrix based on SEED presence/absence gene values was obtained, and the derived triangular matrix was used to obtain a clustering and PCoA analysis where all genomes were distributed accordingly. Additionally, SIMPER analysis was done with the same SEED presence/absence gene values (Table S4) using Bray-Curtis (Table S5). Differences in dispersion of genes were tested by performing an analysis of PERMDISP (107) that includes pairwise comparisons of environments. To test for significant differences between environments, a PERMANOVA was performed using 9,999 permutations. All the calculations were conducted with PRIMER7 software (Primer Ltd., Lutton, UK), and the obtained graph was further edited in Inkscape v.1.0.

Phylogenomic analysis of Gemmatimonadota genomes was done with the PhyloPhlAn 3.0 tool (42, 108). Three genomes from the bacterial phylum Fibrobacterota were used as an outgroup (GCA_900142455.1 *Hallerella intestinalis*, GCA_900217845.1 *Fibrobacter elongatus*, GCA_000146505.1 *F*. *succinogenes*). PhyloPhlAN uses USEARCH (109) to screen for the presence of 400 universally conserved and ubiquitous proteins (found in the PhyloPhlAn database). The alignments of proteins against the built-in database were done using MUSCLE (110), concatenated, and used to generate a maximum-likelihood tree with RAxML (111). The tree was visualized in iTOL (112) and edited using Inkscape v.1.0.

A RuBisCO tree was constructed with representative sequences of the large subunit (*rbcL/cbbL* genes) from various types, including type IA, IB, IC, ID, II, intermediary II/III, III, IV, and archaeal types. This RuBisCO data set was aligned in Geneious Prime (version 2022.2.2) using MAFFT alignment (113, 114) (*n* = 508). Sequences that were not obtained from Gemmatimonadota MAGs were downloaded from UniProt (115) or obtained from previous studies (19, 56, 116, 117). A maximum-likelihood phylogenetic tree was calculated using IQ-TREE (118), with the LG + F + I + G4 substitution model chosen as the best-fitting model by ModelFinder according to the Bayesian Information Criterion (BIC) (119), and 1,000 ultrafast bootstrap replicates. The tree was visualized in iTOL (112) and edited using Inkscape v.1.0.

### Metabolic analysis

This analysis was conducted on genomes with >67% completeness. Gene predictions were performed with PROKKA (120) and diamond (v0.9.14.115), and blastp was used to search versus the KEGG/SEED databases (Table S4). Metabolic features of MAGs were also analyzed with the RAST annotation pipeline database (106) and through BlastKOALA (121), which allowed us to obtain KO identifiers (K numbers) for orthologous genes present in all MAGs (Table S6). Metabolic pathways were then inferred from KEGG (121) and SEED (106) and manually examined for completeness. The percentages of the presence of key genes and pathways were calculated for each environment (Table S6). Plots showing the percentage of the presence of specific metabolic pathways were done in Rstudio (package bubbleplot) and edited in Inkscape 1.0. In Fig. S5, the genomes that had the majority of the genes (>60%) related to flagella were considered to have them present. The figure of metabolic reconstruction was done in Inkscape 1.0, following Table S6.

## Data Availability

All data derived from this work are publicly available in NCBI-GenBank databases. All 16 MAGs assembled in this study have been deposited in the NCBI-GenBank database under Bioproject number PRJNA721863, biosample numbers SAMN32886101-SAMN32886116, and GenBank accession numbers JARIER000000000-JARIFG000000000. All these genomes were derived from metagenomic data sets from Spanish lakes and reservoirs that were previously deposited under Bioproject number PRJNA721863 and SRA numbers SRR15198238- SRR15198275.

## SUPPLEMENTAL MATERIAL

Supplemental Figures spectrum.01112-23-s0001.pdf Fig. S1 to S6.

Table S1 spectrum.01112-23-s0002.xlsx Basic characteristics of newly assembled Gemmatimonadota MAGs.

Table S2 spectrum.01112-23-s0003.xlsx Basic characteristics of all Gemmatimonadota genomes.

Table S3 spectrum.01112-23-s0004.xlsx Percentages of habitat-related core and accessory genes.

Table S4 spectrum.01112-23-s0005.xlsx Presence/absence of genes in Gemmatimonadota based on SEED.

Table S5 spectrum.01112-23-s0006.xlsx SIMPER analysis of similarity and dissimilarity between Gemmatimonadota genomes.

Table S6 spectrum.01112-23-s0007.xlsx Genes and metabolic pathways found in Gemmatimonadota genomes from different environments.

ASM does not own the copyrights to Supplemental Material that may be linked to, or accessed through, an article. The authors have granted ASM a non-exclusive, world-wide license to publish the Supplemental Material files. Please contact the corresponding author directly for reuse.
