# Supplementary material for: Multi-environment ecogenomics analysis of the cosmopolitan phylum Gemmatimonadota
Source: Microbiol Spectr. 2023 Sep 21;11(5):e01112-23. doi: 10.1128/spectrum.01112-23 (PMC10581226; doi:10.1128/spectrum.01112-23)
Supplement: Supplemental Figures — Fig. S1 to S6. [file spectrum.01112-23-s0001.pdf]

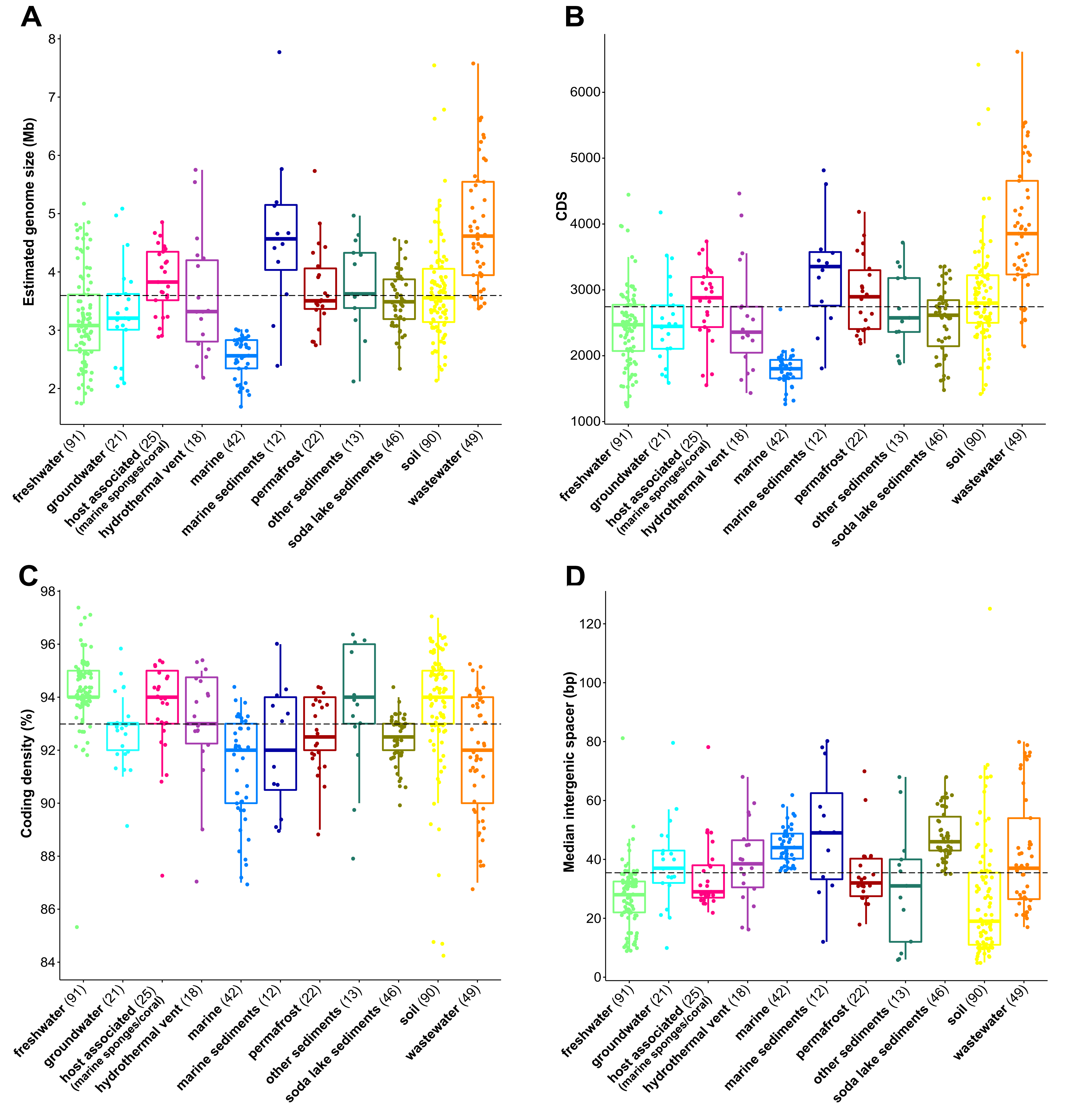


**Figure S1.** Basic characteristics of Gemmatimonadota genomes. Plots show **(A)** range of estimated genome sizes, **(B)** number of CDS, **(C)** ranges of coding density (%) and **(D)** ranges of median intergenic spacer (bp) of Gemmatimonadota genomes from different environments. Environments are color coded, and the number of genomes used in each environment is labeled.

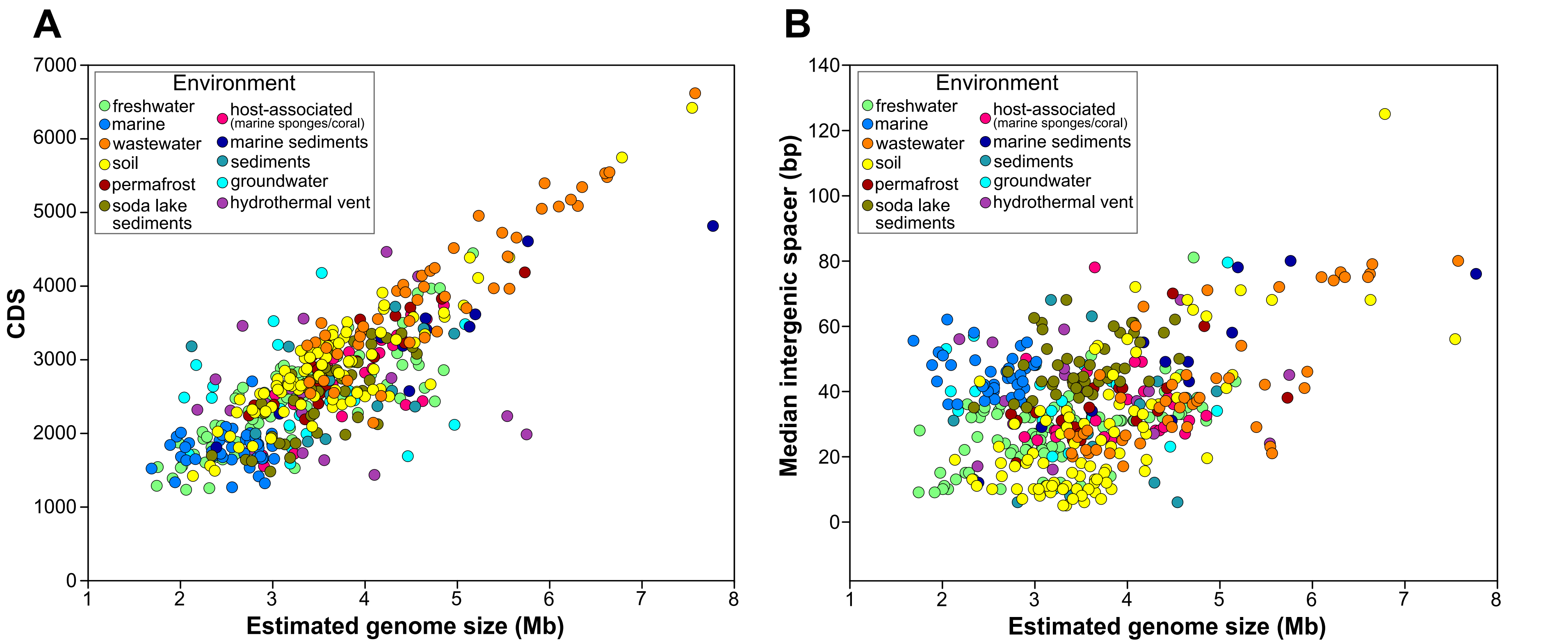


**Figure S2.** Coding density plots showing comparison of **(A)** CDS and **(B)** median intergenic spacer (bp) with the estimated genome size of Gemmatimonadota genomes from different environments color coded.

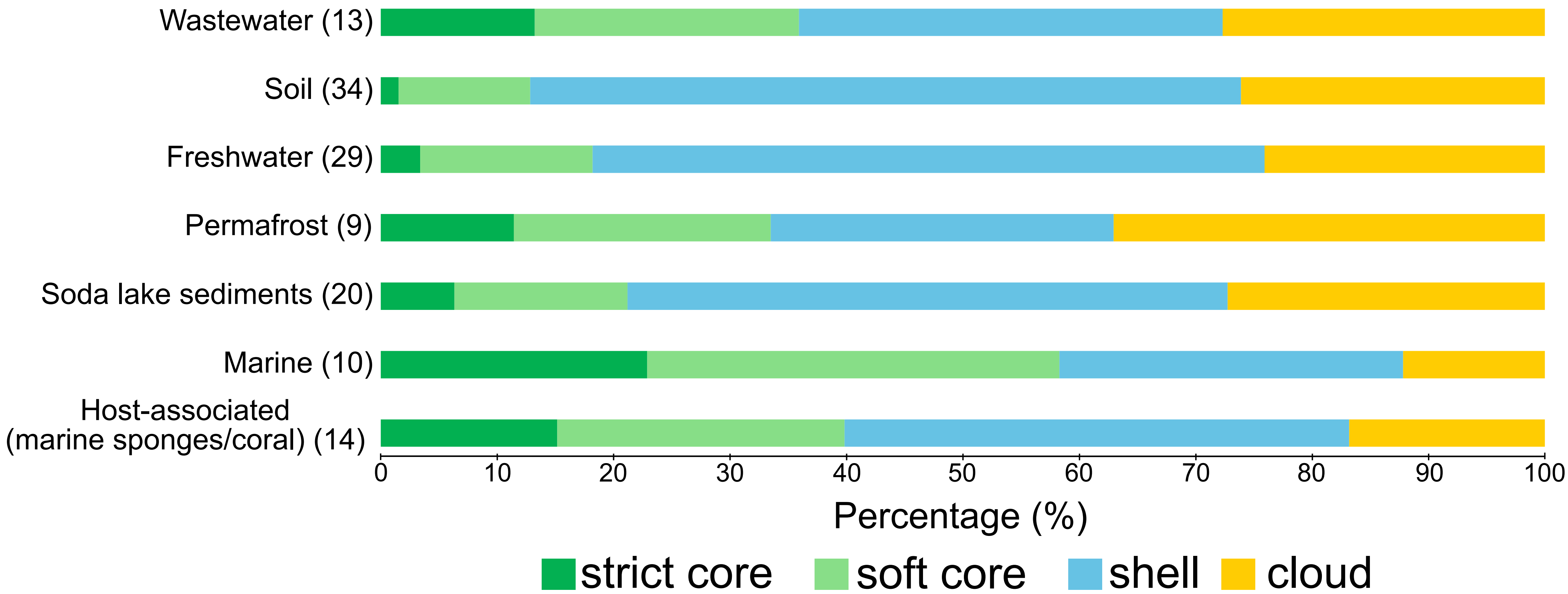


**Figure S3.** Habitat related core and accessory genes of Gemmatimonadota genomes from 7 environments showing percentage of genes forming strict core, soft core, cloud and shell part of the genomes.

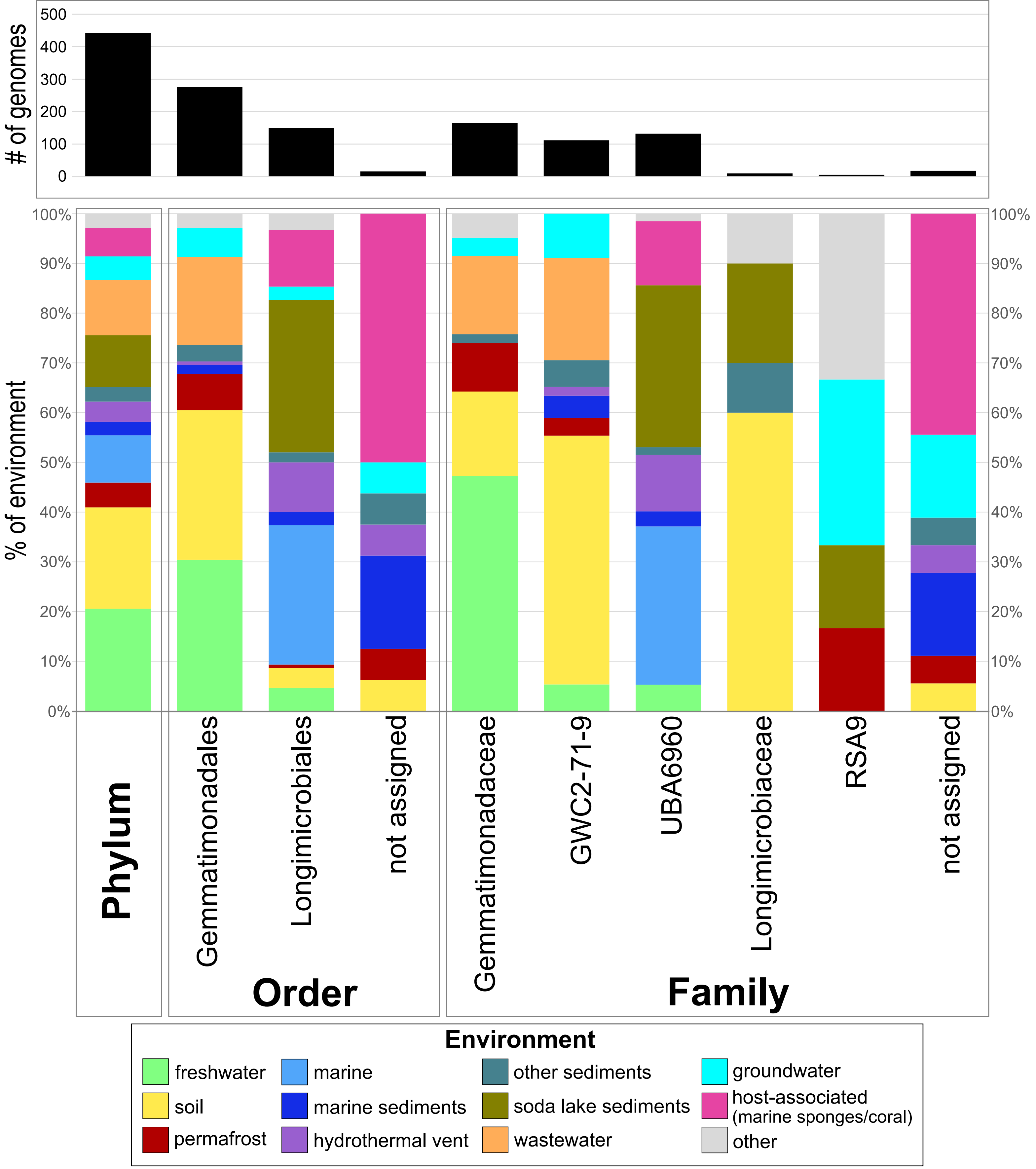


**Figure S4.** Environment related grouping of Gemmatimonadota on different taxonomic levels (phylum, family, order) based on taxonomic assignation by GTDB (101). Bottom legend shows color-coded environments from which the Gemmatimonadota genomes originate.

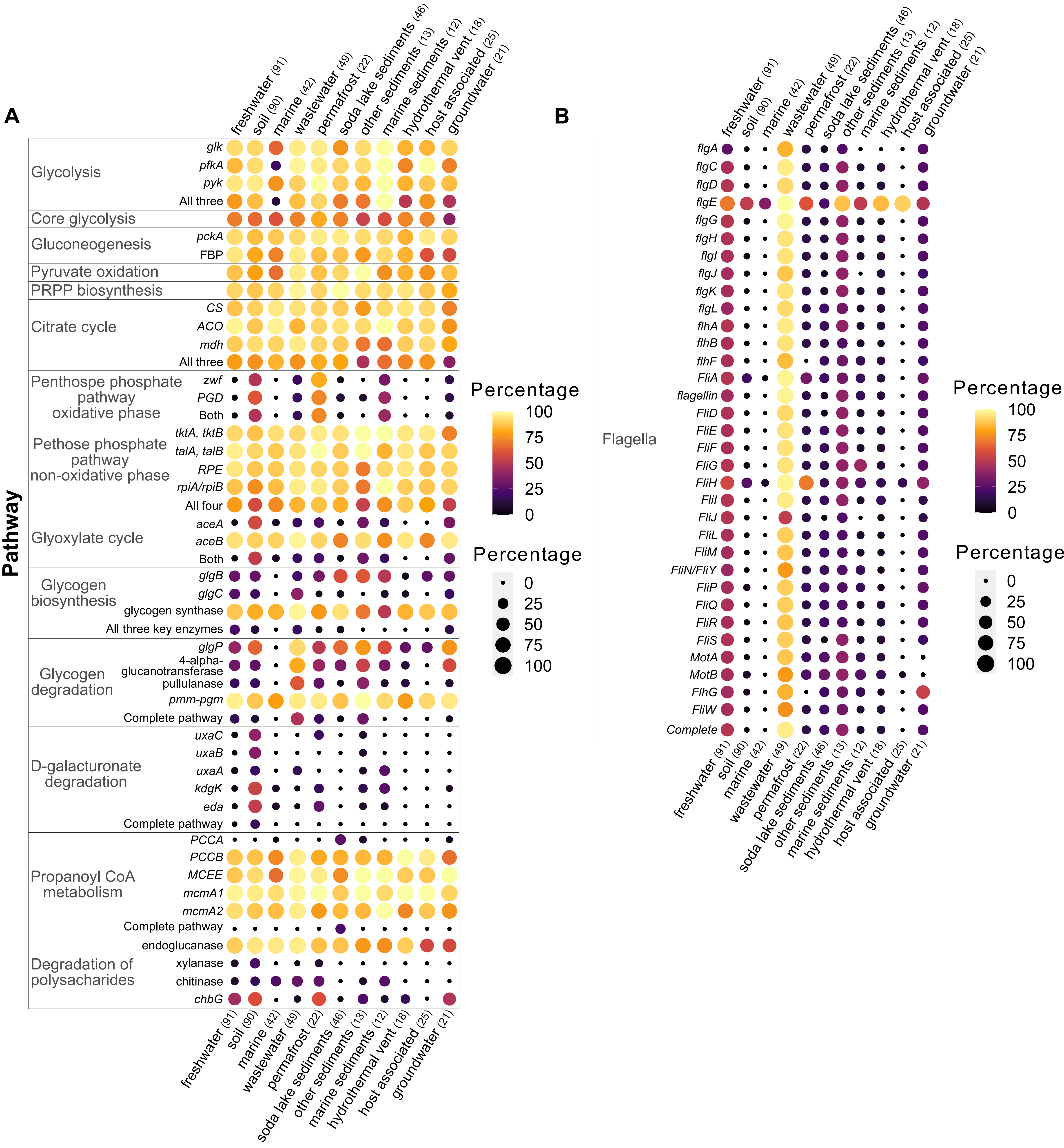


**Figure S5. (A)** Bubble plot showing the percentages of key genes involved in central and other carbohydrate metabolism present in Gemmatimonadota genomes from different environments. **(B)** Bubble plot showing flaggelar assembly genes. Dot color and size indicate the percentage of each gene in certain environment, with darkest color and smallest size of the dot marking the absence of said gene in that environment. The number of MAGs from each environment are labelled in parentheses. Details about gene presence/absence can be found in Table S6.

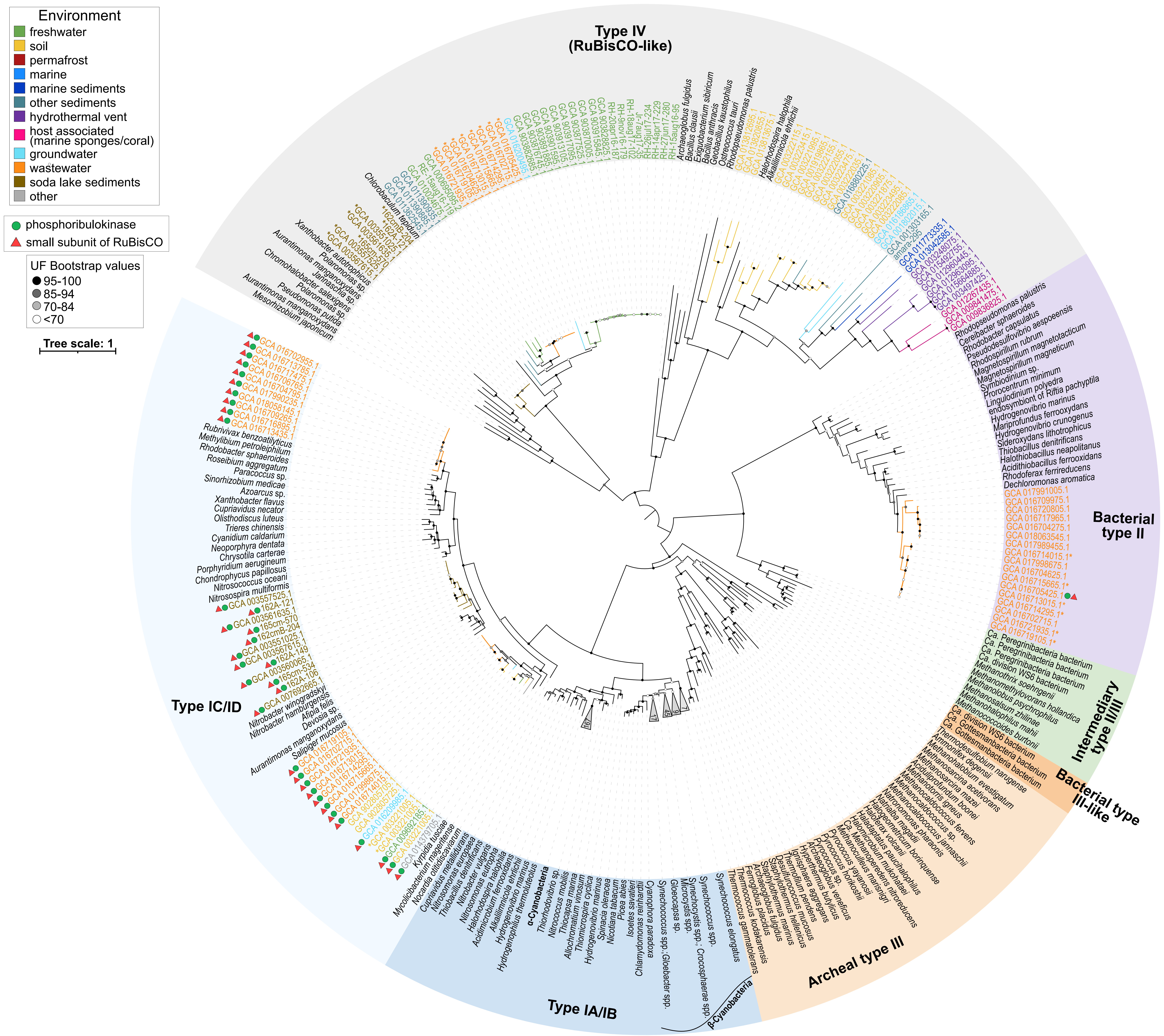


**Figure S6.** Maximum-likelihood phylogenetic tree with (LG+F+I+G4 substitution model chosen as the best-fitting model by ModelFinder according to Bayesian Information Criterion (BIC) (118) and 1000 ultrafast bootstrap replicates) of the large subunit of RuBisCO (types I-III) and RuBisCO like (type IV) proteins (*rbcL*) showing position and classification of Gemmatimonadota RuBisCO sequences. Sequences are color coded based on environment of origin. The presence of small RuBisCO subunit (*rbcS*) and phosphoribulokinase, is labeled with red triangle and green circle, respectively. The numbers shown at collapsed branches (i.e., 7 and 46) indicate the number of genomes (not shown) comprising the respective taxonomic categories.
